# Trust-Based Research: Influencing Factors of Patients’ Medical Choice Behavior in the Online Medical Community

**DOI:** 10.3390/healthcare10050938

**Published:** 2022-05-18

**Authors:** Chutong Qiu, Yuting Zhang, Xiaoyu Wang, Dongxiao Gu

**Affiliations:** 1 Courant Institute of Mathematical Sciences, New York University, New York, NY 10012, USA; cq2039@nyu.edu; 2 School of Economics and Management, Tongji University, Shanghai 200092, China; z_y_t2019@tongji.edu.cn; 3 The Department of Pharmacy, Anhui University of Chinese Medicine, Hefei 230031, China; xywang0551@163.com; 4 The School of Management, Hefei University of Technology, Hefei 230009, China; 5 Key Laboratory of Process Optimization and Intelligent Decision-Making of Ministry of Education, Hefei 230009, China

**Keywords:** online medical community, trust, medical choice behavior, influencing factors

## Abstract

The medical service is a special credit commodity, and trust plays a very important role in patients’ online medical choice behavior. By collecting information about the doctors on China’s leading online medical platform (Platform A), a regression analysis model was constructed, based on the credibility theory model, which has the following three dimensions: ability trust, benevolence trust, and integrity trust. The results showed that the medical title of the doctors, their department’s reputation, the number of gifts given to them, and the number of patients who registered with them after diagnosis, among other factors, had a significant, positive impact on the behavior of choosing doctors. Among these considerations, the number of patients registered after diagnosis had the greatest impact on the behavior of choosing doctors. This factor is the result of each doctor’s personal brand management, which reflects their comprehensive ability, reputation and integrity. Compared with previous studies, this paper creatively analyzed the important influence of departmental reputation and the number of patients registered after diagnosis on medical choice behavior and puts forward that a doctor can use the number of patients registered after diagnosis to manage their personal brand. Based on the results of this study, we will also put forward suggestions from the perspectives of patients, doctors and the online medical community.

## 1. Introduction

With the improvement in living standards and of the health awareness of Chinese residents, and with the intensification of population aging, people’s health needs are becoming more and more vigorous [1]. At the same time, in healthcare, advances in medical technology have brought about some profound changes in trends. First, citizen science is increasingly used in healthcare [2], and it promotes the development of the healthcare ecosystem [3]. Second, the emergence of social media in healthcare has changed the healthcare experience for all involved [4]. All those who are interested in healthcare can obtain and share relevant healthcare information through new channels, such as online healthcare communities, which can even influence their medical decisions [5]. Third, the heightened scrutiny of public health during the COVID-19 pandemic has ignited a host of new research, including supply-side research in the application of new technologies and human resource management. The application of new technologies has facilitated the digital transformation of healthcare, especially after the disruption caused by the COVID-19 pandemic. Telemedicine and digitized medical devices have become a growth area [6] since the pandemic, in order to reach objectives such as relieving the pressure on medical resources, reducing the risk of spreading COVID-19, communicating more conveniently with patients, and of satisfying patients’ need for remote access to healthcare. Furthermore, as an emerging field, cloud healthcare provides a more convenient and effective solution for health [7]. In addition, AI, as an augmentation and assistive technology, has the potential to revolutionize healthcare, including shifting from a therapeutic model to a predictive medical model; providing evidence-based, personalized, and precise treatment [8]; being used for medical decision support [9] so as to free up more time for patient medical treatment; and in constructing smart hospitals and improving the level of healthcare management [10]. In a high-tech healthcare environment, talent retention and management are very important. The high-stakes work environment created by the COVID-19 pandemic has reduced the attractiveness of the healthcare industry and put enormous pressure on staff retention in healthcare organizations, making how to increase the attractiveness of the healthcare industry and avoid staffing shortages a huge challenge that needs to be addressed [11]. Employee job satisfaction and performance could be improved by creating a safer, more pleasant, friendly, and productive environment for employees [12]. The positive emotions of individuals and managers could also have a positive impact on organizational citizenship behavior, which could not only improve organizational performance but may also strengthen employees’ attitudes toward employers and the work environment [13].

The present study extended this line of research from the supply to the demand side of healthcare by focusing on consumers. China’s medical resources suffer from an insufficient supply, an unbalanced structure, and unbalanced distribution, which makes it difficult to meet people’s health needs, making it very difficult to, for example, book a doctors appointment [1]. The online medical community is an example of citizen science in healthcare as well as of preventative healthcare [3,14]. The continuous growth of online medical treatment has made the online medical community develop rapidly as an emerging business form. The online medical community uses information technology to build a medical ecosystem, which includes patients, hospitals, and doctors [15]; it plays an important role in optimizing the allocation of medical resources and in meeting people’s health service needs. It also provides various kinds of health information, such as doctor introductions, treatment experiences, and patient evaluations, that can be used to inform patients’ decision-making and medical choice behavior. Due to the information asymmetry, patients are in information weakness due to the lack of sufficient professional knowledge, which leads to an imbalance between doctors and patients. As a result, making an effective evaluation of doctors’ professional competence and service quality is difficult for patients, which negatively affects their own medical decision making. In addition to medical ability, doctors also need to master the methods needed to demonstrate their professional abilities and service abilities; however, doctors often lack the knowledge and ability to market themselves effectively [9]. As a medical service platform, the online medical community also needs to obtain benefits from the medical behavior of both doctors and patients. The knowledge about how to effectively use the information provided by the online medical community to choose suitable doctors is not only necessary to satisfy the medical needs of patients and the business needs of doctors, but also to facilitate the healthy development of online medical communities. Therefore, based on the data of the online medical platform, Platform A, this paper has studied the influencing factors of patients’ medical choice behavior. One of the aims of this study is to provide reference opinions for patients in the online medical community to help them to choose suitable doctors, and the second is to provide doctors in the online medical community with suggestions that can increase the trust of patients, so that they can provide medical services to more patients. The third aim is to promote the sustainable development of the online medical platform and to enhance the platform’s medical service capabilities.

## 2. Relevant Theories and Hypothetical Models

Trust is an indispensable lubricant in social life and an important condition for equal exchange [16]. Zucher believes that the establishment of trust mechanisms can be based on institutions, characteristics and processes. The characteristics are mainly human attributes, such as family background and ethnicity. The institutions refer to the guarantee of third-party power or to the help of intermediate mechanisms. This process is to trust each other through past or expected exchanges, such as reputation, brand, etc. [17]. McAllister divides trust into cognition-based trust and affect-based trust. Cognition-based trust is based on rational analysis and on the ability judgment of the other party. Affect-based trust is based on goodwill. It is caused by the emotions generated by the interaction between people and has more emotional factors [18]. Sako divides trust into contractual trust, competence trust, and goodwill trust. Contractual trust depends on the contract: the more detailed the contract, the higher the degree of trust. Competence trust is an evaluation of the counterparty’s ability to perform the contract. Goodwill trust is a trust extended out of goodwill, which includes faith, friendship or sympathy [19].

In the network environment, Shankar proposed that online trust includes trust antecedents, online trust, and trust results. The antecedents of trust include influencing factors, such as users, websites, and businesses. Online trust covers, among other factors, credibility, emotional help, skills, and care. The trust results include willingness to use, behavior, and loyalty. [20]. As a special trust commodity [21], trust plays a very important role in the purchase behavior of patients. Trust within the online medical community includes trust in the online medical community itself, the doctors, and the patients. This trust will not only affect users’ decision-making behavior [22], but it will also affect users’ use of the online medical community [23]. Among many trust models, the credibility theoretical model, proposed by Mayer et al., has been widely recognized and applied. This model believes that trust is composed of the following three elements: ability trust, benevolence trust, and integrity trust; it also believes that these three elements can explain most of the contents of trust. Ability trust means that the trusted party has satisfactory professional skills and knowledge, benevolence trust refers to the fact that the trusted party is willing to provide services from the perspective of altruism, and integrity trust means that the trusted party enhances trust by showing real information [24].

Research on trust-based medical choice behavior has emerged. Patients can establish trust relationships through online word of mouth. Doctors’ online reputations can greatly affect patients’ trust and medical choice behavior [25], but it has limitations, such as strong subjectivity, easy distortion, and slow propagation [26]. Doctors can gain the trust of patients by showing their online efforts through active interaction with patients, but there is a lack of evaluation of the doctors’ abilities [27]. However, the activeness of communication between doctors and patients is negatively regulated by a doctor’s medical title and hospital grade [1], as patients often build trust in doctors’ abilities through these two factors [28]. Some studies have evaluated doctors’ abilities by the proportion of the number of patients registered after diagnosis in the total patients [15]. There are also studies that have evaluated benevolence information by the number of articles and the number of services opened, i.e., evaluating the degree of information disclosure through the length of the text content filled in by doctors, in terms of expertise and professional experience, and then establishing integrity trust based on an evaluation of the degree of information disclosure [29]. However, we think that the rationality of this method is questionable. On the basis of previous research, this paper studied the influencing factors of patients’ medical choice behavior in the online medical community, based on the credibility theoretical model proposed by Mayer et al.

### 2.1. Ability Trust and Patients’ Medical Choice Behavior

Medical titles, which are the signal of a doctor’s medical level, are divided into four levels: resident physicians, attending physicians, associate chief physicians, chief physicians. They are evaluated based on the doctors’ education, work experience, and professional achievements, and the evaluation results need for approval from national government departments. Hospital grade is the evaluation of hospital qualifications according to the hospital scale, scientific research direction, talent and technical strength, medical hardware, and equipment: the higher the grade of the hospital, the higher the medical level and the better the doctor resources. Cities are generally classified according to factors such as their level of economic development, and their scale, and cities with higher levels often have better medical resources. The departmental reputation of the doctor’s department is based on the national ranking of the medical and on the academic level of the department. The professional level of the listed hospital represents the top level in this field. The doctors with higher medical titles, higher city grades, and higher departmental reputations, have a higher professional and medical level, which can enhance patients’ trust in the doctor. Therefore, this paper puts forward the following assumptions:

**H1a.**  *Medicaltitle has a significant positive impact on patients’ medical choice behavior*.

**H1b.**  *Hospital grade has a significant positive impact on patients’ medical choice behavior*.

**H1c.**  *City grade has a significant positive impact on patients’ medical choice behavior*.

**H1d.**  *Departmental reputation has a significant positive impact on patients’ medical choice behavior*.

### 2.2. Benevolence Trust and Patients’ Medical Choice Behavior

In the online medical community, doctors can obtain the recognition of consumers through their own efforts, so as to improve their own performance [30]. By publishing articles, doctors popularize health knowledge for patients, so as to improve their contribution value to the online platform. This behavior reflects the doctors’ attention to the online community and their benevolence to patients: the greater the doctor’s effort, the greater the number of articles, the greater the contribution value, and the greater the patient’s benevolence perception. The evaluation of patients’ satisfaction with doctors’ service attitudes reflects the degree of doctors’ benevolence. Factors such as good communication between the doctors and patients, doctors’ abilities to relieve patients’ anxiety and to put themselves in their patients’ shoes, and doctors’ concerns for and affinity with patients can all improve the evaluation of patients’ satisfaction with doctors’ attitudes: the more articles a doctor publishes, the higher the satisfaction with their attitude, the greater the doctor’s degree of benevolence, which helps to improve the trust of patients. Therefore, this paper puts forward the following assumptions:

**H2a.**  *The total number of articles has a significant positive impact on patients’ medical choice behavior*.

**H2b.**  *Attitude satisfaction has a significant positive impact on patients’ medical choice behavior*.

### 2.3. Integrity Trust and Patients’ Medical Choice Behavior

After the diagnosis, patients can be included in the doctor’s post-diagnosis management scope, after their true identity has been verified through the post-diagnosis check-in process. The doctor will then manage the data of the checked-in patients, pay attention to the rehabilitation of these patients, and answer their consultations. The number of patients who successfully check in after diagnosis forms the number of patients who check in after diagnosis, which is an index used to evaluate the comprehensive ability and reputation of the doctors [31]; a good reputation can increase integrity [32]. Doctors guide their patients to check in after their diagnosis through their own efforts. They disclose the real number of patients that they have after diagnosis, in order to show the objective post-diagnosis evaluation information, to reduce information asymmetry, and to enrich patients’ decision-making information. This frank information disclosure behavior can show the doctors’ real comprehensive ability, reputation and integrity [33], so as to form their personal brand influence [34]. This influence is established over time and is therefore more credible and reliable [35] than other indicators. This paper uses the number of patients registered after diagnosis to evaluate the integrity of doctors, and puts forward the following assumptions:

**H3a.**  *The number of patients registered after diagnosis has a significant positive impact on patients’ medical choice behavior*.

### 2.4. Trust Transfer

Gifts, which need to be purchased by patients, are not only the embodiment of doctors’ strength and online reputation, but also an indicator of doctors’ evaluations [34]. Gifts represent doctors’ online recognition [36], which affects users’ purchase behavior through trust transmission [37]. The information asymmetry on Platform A means that the gift factor plays a role in guiding patients’ choice of doctors [38]. This indicator can enhance patients’ trust in doctors [29]. Therefore, this paper puts forward the following assumptions:

**H4a.**  *The number of gifts has a significant positive impact on patients’ medical choice behavior*.

**Figure 1:**
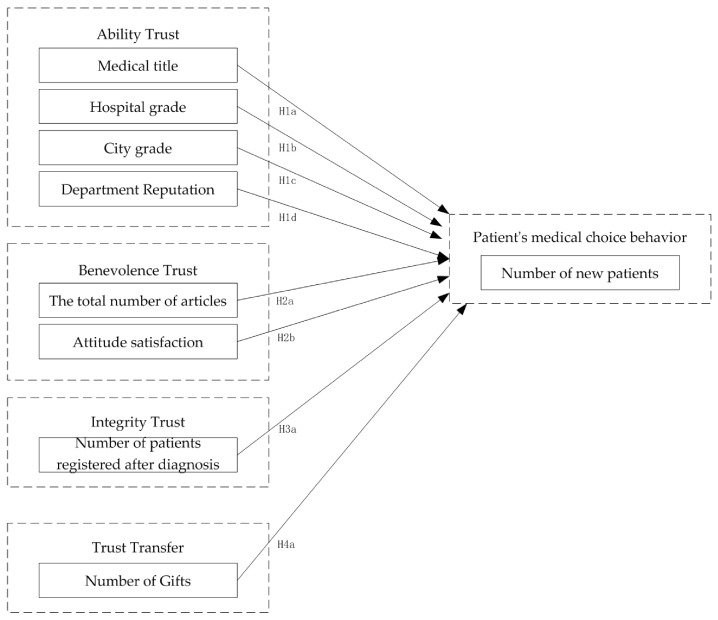
Hypothetical model of influencing factors of patients’ medical choice behavior.

The hypothetical model of the influencing factors of patients’ medical choice behavior was constructed in this study according to the above assumptions. This model is shown in Figure 1.

## 3. Research Design and Data Analysis

### 3.1. Data Collection

The research data came from China’s leading online medical platform, which is presented as “Platform A”. This platform is widely trusted by doctors and patients. As of October 2021, Platform A had a group of 240,000 active doctors, of which doctors in class III hospitals account for 73%, making this platform highly authoritative for medical services. According to the Statistical Bulletin of the People’s Republic of China, on National Economic and Social Development in 2021, issued by the National Bureau of Statistics at the end of 2021, there were 4.27 million licensed doctors and licensed assistant physicians in the country. The number of active doctors on Platform A accounted for 5.62% of the total number of doctors in the country.

This paper collected data on the public information of doctors from 27 departments in January 2022 and March 2022, through Platform A. These departments included obstetrics and gynecology, cardiovascular medicine, pediatrics, cardiovascular surgery, neurology, neurosurgery, respiratory, thoracic surgery, gastroenterology, urology, endocrinology, nephrology, hematology, infection, rheumatology and immunology, orthopedics, ophthalmology, otolaryngology and head and neck surgery, burns, dermatology, plastic surgery, maxillofacial surgery, oncology, vascular surgery, psychiatry, general surgery, and hepatobiliary surgery. The doctors’ information that was collected included medical titles, city names, hospital names, department names, total number of articles, total number of patients, number of patients registered after diagnosis, efficacy satisfaction, attitude satisfaction, and the number of thank you letters and the number of gifts that they received. All the data were desensitized and contained no privacy violations. After data cleaning, 37,341 sample data were finally obtained.

### 3.2. Variable Definition

The dependent variable of this study was the number of new patients. The independent variables were the doctor’s title, the total number of articles published, attitude satisfaction, the number of patients registered after diagnosis, and the number of gifts received. The control variables were the city grade, the hospital grade, and the departmental reputation. The variables are explained as follows:

1) Medical title (Mtitle)

Since chief physicians, associate chief physicians, and attending physicians account for the absolute majority among doctors, we coded the chief physicians as four, the associate chief physicians as three, the attending physicians as two, and the physicians of other grades as one.

2) Hospital grade (Hospital)

Since the highest-grade hospitals account for the absolute majority, we coded the highest-grade hospitals as one, and the rest as zero.

3) City grade (City)

This article drew on the classification of cities in the “2021 City Commercial Charm Ranking”, released by China Business News, and the codes were as follows: six for first-tier cities, five for new first-tier cities, four for second-tier cities, three for third-tier cities, two for fourth-tier cities, one for fifth-tier cities, and zero for other cities.

4) Department Reputation (Reputation)

According to the ranking of specialized fields in the 2021 ranking list of Internet influence of Chinese hospitals, released by the health industry development research center of the Chinese Academy of Social Sciences, in November 2021, the code of departments on the list is one, and the code of departments not on the list is zero.

5) The total number of articles (N_articles)

The total number of articles = the cumulative number of articles published by doctors as of March 2022.

6) Attitude satisfaction (A_satisfac)

Attitude Satisfaction = Attitude Satisfaction Rating for March 2022.

7) Number of patients registered after diagnosis (N_Afterdia)

The number of patients registered after diagnosis is the cumulative number of patients registered after diagnosis as of March 2022.

8) Number of Gifts (Gifts)

The number of gifts is the cumulative number of gifts received by doctors as of March 2022.

9) Efficacy satisfaction (E_satisfac)

Efficacy satisfaction = efficacy satisfaction evaluation in March 2022.

10) Number of thank you letters (Letters)

The number of thank you letters is the cumulative number of thank you letters received by doctors as of March 2022.

11) Number of new patients (N_patients)

In this paper, the number of new patients in a certain period of time was selected to reflect the patients’ medical choice behavior, so the calculation formula of medical choice behavior was as follows:

Number of new patients = total number of patients in March 2022–total number of patients in January 2022.

### 3.3. Descriptive Statistics and Correlation Analysis

After the descriptive statistical analysis of the cleaned data, it is concluded that the data skewness of the total number of articles, the number of patients registered after diagnosis, the number of gifts, and the number of new patients was greater than three, which needed to be processed by logarithmic conversion. The descriptive statistical analysis after logarithmic conversion is shown in Table 1, and the skewness of variables was less than three.

The correlation analysis of variables is shown in Table 2. The results show that the correlation coefficients between variables are within a reasonable range.

### 3.4. Model Construction

In this paper, Stata software was used for the multiple regression analysis to verify the research hypothesis. The research model is as follows:

In(N_patients) = *β*_0_ + *β*_1_Hospital + *β*_2_City + *β*_3_Reputation + *ε* (1)

In(N_patients) = *β*_0_ + *β*_1_Hospital + *β*_2_City + *β*_3_Reputation + *β*_4_Mtitle + *β*_5_A_satisfac + *β*_6_ln(N_articles) + *ε* (2)

In(N_patients) = *β*_0_ + *β*_1_Hospital + *β*_2_City + *β*_3_Reputation + *β*_4_Mtitle + *β*_5_A_satisfac + *β*_6_ln(N_articles) + *β*_7_ln(N_Afterdia) + *β*_8_(ln(Gifts) + *ε* (3)

where, *β*_i_ (i = 0, 1 … 9) represents the parameter to be estimated; 𝜀 is the error term.

Only the control variables were put into Model 1, the attitude satisfaction and the total number of articles published were put into Model 2, and all the variables were put into Model 3.

### 3.5. Results Analysis

The grouping regression results are shown in Table 3. We also tested the variable variance expansion factor (VIF). The VIF value of each model was less than five and the mean value was less than three. As a result, there were no serious multicollinearity problems in the model.

In Model 1, the hospital grade, city grade, and departmental reputation were significant at the 1% level, and the coefficients were positive, indicating that these variables can significantly and positively affect patients’ medical choice behavior. Among them, the departmental reputation had the largest reputation coefficient and influence, which also reflects that the listed departments are at the top level in their specialty fields.

According to the results of Model 2, the hospital grade, city grade, departmental reputation, medical title, attitude satisfaction, and the number of published articles were significant at the level of 1%, and the coefficients were positive. After adjustment, R-squared increased significantly, indicating that it had a good fitting degree, and that the introduced variables had a strong explanatory effect on the dependent variables. The hospital grade, city grade, and departmental reputation can have a significant positive impact on patients’ medical choice behavior, in terms of ability trust and benevolence trust. The coefficients of departmental reputation and medical title in the variables were far from those of other variables, indicating that patients are more inclined to choose appropriate doctors from the ability dimensions of departmental reputation and medical title, in the absence of more decision-support information.

Model 3 was the main research model of this paper. It introduced the number of patients registered after diagnosis and the number of gifts to Model 2. After adjustment, the R-squared increased slightly and the fitting degree was good. The coefficients of all the variables were positive: the hospital grade was not significant, the city grade was significant at the level of 10%, and the other variables were significant at the level of 1%. Therefore, it was assumed that H1b is not tenable, but that H1a, H1c, H1d, H2a, H2b, H3a, and H4a are tenable. The coefficients of hospital grade, city grade, departmental reputation and medical title were positive, which means these factors have a positive impact on medical choice behavior. These reflect that those doctors with a higher city grade, a higher hospital grade, a higher departmental reputation, and a higher medical title, have a higher professional and medical level, which can enhance patients’ trust in doctors. The reputation of the department represents that the hospital is at the top level in its specialty field, and this factor has the greatest influence on patients’ medical choice behavior. In reality, each hospital may have key specialty fields, such as provincial and municipal clinical key specialties. Most of the doctors on Platform A are chief physicians. Under the same medical title, due to the different expertise of each doctor, the medical skills are also different; in some characteristic key-specialty fields, doctors with low-level medical titles may also have a high medical level. For example, the medical skills of the associate chief physicians of the provincial clinical key specialty of a hospital may surpass those of the chief physicians of other hospitals at the same level. Doctors’ positive personal efforts, such as the number of published articles, and other attitude satisfaction factors, can reflect their benevolence and win the trust of patients. Because the gifts of the online medical community need to be purchased, the number of gifts can also reflect the reputation of doctors. The number of patients registered after diagnosis and the number of gifts have a significant positive impact on patients’ medical choice behavior and can also weaken the influence of the hospital and city grades. Since the number of patients registered after diagnosis needs to be accumulated over time, it has higher reliability and more important evaluation value.

### 3.6. Robustness Test

Since both letters and gifts are the forms in which patients express their gratitude to the doctors, the correlation coefficient between doctors’ efficacy satisfaction and attitude satisfaction is 0.99. In order to verify the robustness of the model, this paper used letters of thanks to replace gifts, and efficiency satisfaction to replace attitude satisfaction. The regression results are shown in Table 4. The regression results show that the results of Model 2 are consistent with the previous model. The coefficients in the results of Model 3 were positive, which is consistent with the previous model. The city grade changed from 10% to non-significant, and the significance of other variables remained unchanged. From the above analysis, it can be concluded that the results of this study are robust.

## 4. Discussion

Based on trust theory, this paper studied the influencing factors of patients’ medical choice behavior in the online medical community. Through an analysis of the data collected from Platform A in January 2022 and March 2022, the research results show that most assumptions are supported. The research recommendations are as follows:

(1) When choosing a doctor, patients need to evaluate the medical skills of each doctor from multiple angles, especially to understand the specialty of the doctor and the specialty level of the department. They also need to give priority to doctors from departments with a higher departmental reputation grade. Patients should also pay attention to the credibility of the doctor, as implied by the number of patients registered after diagnosis: the higher the number, the higher the credibility of the doctor. Patients should also evaluate the doctor’s comprehensive ability, reputation and integrity according to the number of patients registered after diagnosis and the number of gifts and choose a doctor with brand influence.

(2) Doctors should conduct personal brand management through the number of patients registered after diagnosis, thus enhancing their personal reputation and gaining integrity recognition. Doctors should also upgrade their skills and have higher-level medical titles. Furthermore, they should disclose as much personal attribute information as possible, such as education, medical skills, and specialties to enhance their integrity. The positive personal efforts of doctors, such as publishing articles and improving service attitudes, can reflect their own benevolence and gain the trust of patients.

(3) As an online medical community, Platform A should show detailed properties of hospitals and doctors, such as the specialist capabilities of hospital departments, doctors’ education and medical skills, etc. Since the patient satisfaction evaluation on it is not perfect, we suggest that the online medical community needs to improve the patient satisfaction evaluation mechanism so as to enable patients to obtain more accurate evaluation information.

Compared with the research of other scholars, the creativity of this paper lies in the following two points:

(1) The reputation of the doctors’ departments were included in the influencing factors, and the research results showed that this has a very important influence on patients’ choices. When choosing a doctor, patients need to evaluate the medical skills of each doctor from multiple angles, especially to understand the specialty of the doctor and the specialty level of their department.

(2) We propose that the number of patients registered after diagnosis is the result of doctors’ efforts. This type of frank information disclosure behavior can show patients the real comprehensive ability, reputation and integrity of a doctor, and is more credible and reliable than other factors. Doctors can use this influencing factor to operate personal brands.

There are still deficiencies in this study. Because the data only came from Platform A, the applicability of these research results may face uncertainty in the face of other online medical community data sources. In addition, due to the limited data information of Platform A, the research results have certain limitations.

## Figures and Tables

**Table 1:** Descriptive statistics of variables.

Variables	Quantity	Standard Deviation	Mean	Max	Min	Skewness
Mtitle	37,341	0.697	3.452	4	1	−1.077
Hospital	37,341	0.282	0.913	1	0	−2.924
City	37,341	1.698	4.361	6	0	−0.990
Reputation	37,341	0.318	0.114	1	0	2.429
N_patients	37,341	1.250	0.559	7.509	0	2.353
A_satisfac	37,341	34.488	13.981	100	0	2.065
N_articles	37,341	1.411	0.910	8.865	0	1.475
N_Afterdia	37,341	2.534	1.973	10.721	0	1.029
Gifts	37,341	1.916	1.390	9.198	0	1.237

**Table 2:** Correlation analysis results.

Variables	1	2	3	4	5	6	7	8	9
1 N_patients	1.000								
2 Mtitle	0.123	1.000							
3 Hospital	0.094	0.101	1.000						
4 City	0.260	0.068	0.113	1.000					
5 Reputation	0.277	0.053	0.102	0.357	1.000				
6 A_satisfac	0.686	0.061	0.073	0.214	0.240	1.000			
7 N_articles	0.422	0.079	0.033	0.172	0.119	0.399	1.000		
8 N_Afterdia	0.600	0.133	0.116	0.266	0.259	0.584	0.522	1.000	
9 Gifts	0.607	0.142	0.122	0.330	0.292	0.557	0.606	0.785	1.000

**Table 3:** Analysis of regression results.

Variables	Model (1)		Model (2)		Model (3)	
	Coef.	VIF	Coef.	VIF	Coef.	VIF
Hospital	0.151 ***	1.03	0.055 ***	1.04	0.001 NS	1.05
City	0.107 ***	1.13	0.026 ***	1.16	0.004 *	1.19
Reputation	0.962 ***	1.11	0.405 ***	1.15	0.263 ***	1.19
Mtitle			0.601 ***	1.02	0.030 ***	1.03
A_satisfac			0.023 ***	1.30	0.018 ***	1.95
N_articles			0.143 ***	1.24	0.028 ***	1.68
N_Afterdia					0.054 ***	4.02
Gifts					0.150 ***	4.38
constant	−0.154 ***		−0.313 ***		−0.182 ***	
R-squared	0.107		0.590		0.629	
Number of obs	37,341		37,341		37,341	
Mean VIF	1.09		1.15		2.06	

Note: *** indicates *p* < 0.01, * indicates *p* < 0.1, NS: not significant.

**Table 4:** Analysis of regression results.

Variables	Model (1)		Model (2)		Model (3)	
	Coef.	VIF	Coef.	VIF	Coef.	VIF
Hospital	0.151 ***	1.03	0.057 ***	1.04	0.001 NS	1.05
City	0.107 ***	1.13	0.026 ***	1.16	0.001 NS	1.23
Reputation	0.962 ***	1.11	0.408 ***	1.15	0.240 ***	1.23
Mtitle			0.061 ***	1.02	0.020 ***	1.04
E_satisfac			0.023 ***	1.29	0.018 ***	2.08
N_articles			0.144 ***	1.24	0.057 ***	1.51
N_Afterdia					0.073 ***	3.71
Letters					0.150 ***	4.35
constant	−0.154 ***		−0.317 ***		−0.152 ***	
R-squared	0.107		0.586		0.625	
Number of obs	37,341		37,341		37,341	
Mean VIF	1.09		1.15		2.02	

Note: *** indicates *p* < 0.01, NS: not significant.

## Data Availability

The image data used to support the findings of this study are available from the corresponding author upon request.
